# Spontaneous generation of diversity in *Arachis* neopolyploids (*Arachis ipaënsis* × *Arachis duranensis*)^4x^ replays the early stages of peanut evolution

**DOI:** 10.1093/g3journal/jkab289

**Published:** 2021-08-31

**Authors:** Soraya C M Leal-Bertioli, Eliza F M B Nascimento, M Carolina F Chavarro, Adriana R Custódio, Mark S Hopkins, Márcio C Moretzsohn, David J Bertioli, Ana Claudia G Araújo

**Affiliations:** 1 Institute of Plant Breeding, Genetics and Genomics, Athens, GA 30602-6810, USA; 2 Department of Plant Pathology, University of Georgia, Athens, GA 30602, USA; 3 Embrapa Genetic Resources and Biotechnology, Brasília, 70770-917, Brazil; 4 Institute of Biological Sciences, University of Brasilia, Brasília, 70910-000, Brazil; 5 Department of Crop and Soil Science, University of Georgia, Athens, GA 30602-6810, USA

**Keywords:** *Arachis*, cytogenetics, domestication, genome instability, homoeologous recombination, neopolyploids, SNPs, polyploidy, morphology

## Abstract

Polyploidy is considered a driving force in plant evolution and domestication. Although in the genus *Arachis*, several diploid species were traditionally cultivated for their seeds, only the allotetraploid peanut *Arachis hypogaea* became the successful, widely spread legume crop. This suggests that polyploidy has given selective advantage for domestication of peanut. Here, we study induced allotetraploid (neopolyploid) lineages obtained from crosses between the peanut’s progenitor species, *Arachis ipaënsis* and *Arachis duranensis*, at earlier and later generations. We observed plant morphology, seed dimensions, and genome structure using cytogenetics (FISH and GISH) and SNP genotyping. The neopolyploid lineages show more variable fertility and seed morphology than their progenitors and cultivated peanut. They also showed sexual and somatic genome instability, evidenced by changes of number of detectable 45S rDNA sites, and extensive homoeologous recombination indicated by mosaic patterns of chromosomes and changes in dosage of SNP alleles derived from the diploid species. Genome instability was not randomly distributed across the genome: the more syntenic chromosomes, the higher homoeologous recombination. Instability levels are higher than observed on peanut lines, therefore it is likely that more unstable lines tend to perish. We conclude that early stages of the origin and domestication of the allotetraploid peanut involved two genetic bottlenecks: the first, common to most allotetraploids, is composed of the rare hybridization and polyploidization events, followed by sexual reproductive isolation from its wild diploid relatives. Here, we suggest a second bottleneck: the survival of the only very few lineages that had stronger mechanisms for limiting genomic instability.

## Introduction

Polyploidy is near universal in plants: over evolutionary time, all, or almost all angiosperms have undergone at least one polyploidization event, also known as a whole-genome duplication (Wendel 2015). In addition, about half of all higher plant species have undergone more recent polyploidization (Soltis *et al.* 2015). Polyploidy has long been viewed as advantageous: it provides heterosis and gene redundancy and brings enhanced novelty that can broaden phenotypic plasticity providing the ability to adapt to new ecological niches (Comai 2005; Soltis *et al.* 2015; Blaine Marchant *et al.* 2016; Soltis and Soltis 2016; Van de Peer *et al.* 2017). Crosses between individuals with different ploidy levels usually result in sterile offspring, therefore, polyploidy precipitates speciation between the new polyploid and its associated progenitor diploids (Ramsey and Schemske 1998; Adams and Wendel 2005). Two main types of polyploidy can be identified: autopolyploidy, the duplication of the chromosome set of one species, and allopolyploidy, the merger of genomes from different species into a single nucleus, and whole-genome duplication (Stebbins 1971; Wendel and Doyle 2005; Mason and Wendel 2020). The initial stage of allopolyploidization, involving sudden changes due to the merge of different genomes, has been classically known as “genetic shock” (McClintock 1984). This includes genetic, epigenetic, and gene expression changes. These changes can occur rapidly, as early as in somatic cells of interspecific F_1_ hybrids, or in the generations following chromosome doubling. Examples of successful allopolyploid modern species are rapeseed, coffee, tobacco, cotton, and peanut (Kochert *et al.* 1996; Leitch *et al.* 2008; Yoo *et al.* 2013; Chalhoub *et al.* 2014; Lashermes *et al.* 2014).

The allotetraploid peanut (*Arachis hypogaea* L.) belongs to the genus *Arachis* that has 83 described species, divided into nine taxonomical sections (Valls and Simpson 2005, 2017; Krapovickas and Gregory 2007; Valls *et al.* 2013; Santana and Valls 2015; Seijo *et al.* 2021). Peanut belongs to the section *Arachis*. The section has six recognized genome types, based on the distribution patterns of heterochromatic bands and rDNA loci: A, B, D, F, G, and K-genome types, with two basic chromosome numbers (*x* = 10 and *x* = 9), and two ploidy levels (2x and 4x) (Smartt and Gregory 1967; Stalker 1991; Seijo *et al.* 2004; Robledo and Seijo 2008, 2010; Robledo *et al.* 2009; Silvestri *et al.* 2015). Peanut is an allotetraploid with an AABB type genome, 2*n* = 4x = 40 (Husted 1936), that arose from one or a very few events of hybridization and tetraploidization (Kochert *et al.* 1996; Bertioli *et al.* 2019). Cytogenetic, phylogeographic and molecular evidence indicate *Arachis* *duranensis* Krapov. & W.C. Greg. and *Arachis* *ipaënsis* Krapov. & W.C. Greg. as the A and B subgenomes parents of peanut, respectively (Kochert *et al.* 1996; Seijo *et al.* 2004, 2007; Ramos *et al.* 2006; Robledo *et al.* 2009; Lavia *et al.* 2011; Grabiele *et al.* 2012; Moretzsohn *et al.* 2013). These two species diverged 2–3 Mya (Moretzsohn *et al.* 2013; Bertioli *et al.* 2016). Hybridization of *A. duranensis* and *A. ipaënsis*, spontaneous polyploidy (about 5000 and 10,000 years ago), and domestication gave rise to the modern crop (Bertioli *et al.* 2016, 2019, 2020). The initial genome duplication event isolated the new allopolyploid from all other diploid *Arachis* species. DNA evidence indicates a very narrow polyploid origin that generated a significant bottleneck (Bertioli *et al.* 2019; Yin *et al.* 2020). Despite this, peanut has evolved into diverse growth habits, architecture, and morphological forms; shorter and longer growing seasons; different testa colors and numbers of seeds per pod, and different hues and patterning of flowers. Two subspecies of *A. hypogaea* are recognized, *hypogaea* and *fastigiata* between which, remarkably, there is partial sexual incompatibility. The two subspecies have two (*hypogaea* and *hirsuta*) and four (*fastigiata*, *vulgaris*, *aequatoriana*, and *peruviana*) botanical varieties, respectively (Krapovickas and Gregory 2007). Even though several diploid *Arachis* species were cultivated before the tetraploid, only the latter fully developed a domestication syndrome and was dispersed around the globe to become a crop of worldwide importance (See Supplementary Note 1 in Bertioli *et al.* 2019). This strongly implicates polyploidy as a significant factor for niche expansion into domestication and subsequent success as a crop plant, perhaps driven by increased evolutionary phenotypic plasticity, and ability to adapt to different environments. Here, we investigated induced allotetraploids generated from peanut’s progenitor species with the rationale that they likely replay the initial genetic events following the origin of *A. hypogaea* and its wild counterpart of common origin, the allotetraploid *Arachis* *monticola*.

Both progenitor species are extant and were recently sequenced (Bertioli *et al.* 2016). The B genome donor, *A. ipaënsis*, is nowadays represented by only a single accession, K 30076. Biogeography indicates that it was moved by humans in prehistory into the range of the A subgenome donor, thus enabling the formation of the allotetraploid species. Extraordinary DNA identity (modal values of 99.98%) between *A. ipaënsis* and the B subgenome of peanut indicate that it likely descended from the very same population that gave rise to peanut in a very recent polyploidy event (Bertioli *et al.* 2016; Yin *et al.* 2020). The sequenced representative of the A subgenome donor species, *A. duranensis* V 14167, is also very close to the A subgenome of cultivated peanut, with modal identity around 99.75% (Bertioli *et al.* 2020). The availability of such close representatives of the ancestors of cultivated peanut provides us with remarkable opportunities to “replay” the origin of *A. hypogaea*. Here, we used lineages from two independent induced polyploidy events (Favero *et al.* 2006; Bertioli *et al.* 2019), spanning 11 generations. These induced allopolyploids spontaneously generated phenotypic and genome diversity, in a way that likely mimics events that occurred between about 5000 and 10,000 years with the spontaneous origin of tetraploid peanut: the first bottleneck, the polyploidization, that isolated the original allotetraploid from the wild diploid relatives, and the second bottleneck proposed here, is the control of genomic instability allowing survival of few lineages.

## Materials and methods

### Plant material

Lineages from two independent induced allotetraploids (neopolyploids) were used in this study, both derived from crosses between accessions of the peanut’s progenitor species *A. ipaënsis* K 30076 (female), and *A. duranensis* V 14167 (male). These were also the accessions used to construct the diploid reference genomes (Bertioli *et al.* 2016). Although all evidence points to *A. duranensis* being the female progenitor (Kochert *et al.* 1996; Grabiele *et al.* 2012), its delicate flower and other unknown factors have prevented any AB hybrid from being produced, with a unique exception, very recently published (García *et al.* 2020). Diploid BA hybrids were treated with colchicine for chromosome doubling, as described in Leal-Bertioli *et al.* (2017). Lineages from the neopolyploid obtained by Favero *et al.* (2006) are here termed IpaDur1. IpaDur1 was propagated and maintained for nine generations of self-pollination, without intentional selection, regardless of morphological aspects, pollen viability, or fertility. The neopolyploid obtained years later using the same parents (Bertioli *et al.* 2019) is here termed IpaDur2. Different independent lineages of two generations (S_2_ and S_3_) from a single hybrid IpaDur2 were studied individually. These and all other genotypes used in this study are listed on Table 1.

**Table 1:** Wild, cultivated, and induced allotetraploid *Arachis* genotypes used for phenotypic, cytogenetic, and genotypic analyses

Genotype	Plant ID	Ploidy	Genome type	Collection site
**Wild species**				
*A. duranensis* Krapov. & W.C. Greg.	V 14167	2x	AA	Salta, Argentina
*A. ipaënsis* Krapov. & W.C. Greg.	K 30076	2x	BB	Gran Chaco, Bolivia
*A. monticola* Krapov. & Rigoni	V 14165	4x	AABB	Jujuy, Argentina
**Cultivated peanut**	**Plant ID**	**Ploidy**	**Genome type**	**Classification**
*A. hypogaea* L. subsp. *fastigiata* Waldron var. *fastigiata*	“IAC-Tatu-ST”	4x	AABB	Modern cultivar
**Induced allotetraploids**	**Plant ID**	**Ploidy**	**Genome type**	**Classification**
[*A. ipaënsis* K30076 **×** *A. duranensis* V14167]^4x^	IpaDur1*a*	4x	BBAA	Induced allotetraploid at generations S_1_ to S_10_
[*A. ipaënsis* K30076 **×** *A. duranensis* V14167]^4x^	IpaDur2*b*	4x	BBAA	Induced allotetraploid at generations S_2_ and S_3_

a Favero *et al.* (2006).

b Bertioli *et al.* (2019).

### Plant phenotyping

In order to investigate phenotypic variation between different allotetraploids produced from the same diploid hybrid, general traits were observed (plant habit, main stem height, and leaf size) and seed width and length were measured from 16 IpaDur2 plants at S_2_ (second generation of self-pollination). For comparison, *A. duranensis* and *A. ipaënsis* seeds were also measured. Data were analyzed using the Shapiro–Wilk test for normality. For comparing groups, ANOVA (parametric) or Kruskal–Wallis test (nonparametric) were used. Variance of each group was compared using the Levene’s test for equality of variances (Levene 1960). All tests and plots were performed using R package. The color of the flowers of the 16 IpaDur2 S_2_ plants was determined at onset of anthesis.

### Cytogenetic analysis

#### Chromosome preparations:

Cytogenetic analyses were performed on samples of both induced allotetraploids, IpaDur1 S_10_ (10th generation of self-pollination), IpaDur2 S_2_ (2nd generation of self-pollination). Root tips were isolated from at least five plants of each IpaDur2 and IpaDur1. At least 10 metaphases of each plant (over 250 metaphases per genotype) were observed. Root tips (5–10 mm long) from 4-week-old plants were collected, treated with 2 mM 8-hydroxyquinoline for 2 h at room temperature and another hour at 4^°^C with new solution, and then incubated in fixative solution (absolute ethanol: glacial acetic acid, 3:1, v/v) for 12 h at 4°C. Somatic chromosome spreads were prepared according to Schwarzacher and Heslop-Harrison (2000) with few modifications: meristems were digested in 10 mM citrate buffer containing 2% cellulase (from *Trichoderma viridae*; Onozuka R-10 Serva) and 20% pectinase (from *Aspergillus niger*, Sigma) for 2 h at 37°C. Chromosomes of each root were set on a slide, in a drop of acetic acid 45% and the spread was obtained by applying pressure to coverslip. Slides were selected using phase contrast in the AxiosKop microscope (Zeiss, Oberkochen, Germany). Coverslips were removed, slides were air-dried for 24 h and kept at −20°C.

#### CMA3+/DAPI heterochromatic banding

${\text{CMA}}_{3}^{+}$ /DAPI banding was conducted with IpaDur1 S_10_ and IpaDur2 S_2_ plants following (Schweizer and Ambros 1994) to localize GC and AT-rich heterochromatic regions, respectively. Chromosome spreads were treated with 0.5 mg/ml of chromomycin A3 (${\text{CMA}}_{3}^{+}$) for 1 h at room temperature, then with 2 µg/ml of 4', 6-diamino-2-phenylindole (DAPI) for 30 min at room temperature. Slides were mounted with glycerol/McIlvaine buffer and analysis was conducted in the epifluorescence Zeiss AxioPhot photomicroscope (Zeiss, Oberkochen, Germany). Images were captured using Zeiss AxioCam MRc digital camera (Zeiss Light Microscopy, Göttingen, Germany) and AxioVision Rel. 4.8 software and further processed using the Adobe Photoshop CS10 software, applying only functions that affect the whole image equally. At least 50 metaphases of each plant (250 metaphases per genotype) were observed.

### In situ hybridization—FISH and GISH

#### Production of probes

Probes for genomic *in situ* hybridization (GISH) were obtained from genomic DNA (1 μg) isolated from young leaflets of *A. duranensis* and *A. ipaënsis*, using a CTAB protocol (Doyle and Doyle 1987). Purified DNA (1 μg) was labeled with either, digoxigenin-11-dUTP (Roche Diagnostics Deutschland GmbH) or Cy3-dUTP (Roche Diagnostics Deutschland GmbH) by Nick Translation kit (Roche Diagnostics Deutschland GmbH). For fluorescent *in situ* hybridization (FISH), we used clones containing the sequences corresponding to 5S ribosomal DNA of *Lotus japonicus* (Pedrosa *et al.* 2002) and 18S-5.8S-25S of *Arabidopsis thaliana* (Wanzenböck *et al.* 1997). The rDNA was isolated with the Illustra plasmid Prep Midi Flow kit (GE Heltcare) and rDNA sequences were labeled by Nick translation, using the same kits as described for the genomic probes.

#### Hybridization

The *in situ* hybridization experiments were performed as described by Schwarzacher and Heslop-Harrison (2000). Hybridization steps and conditions were identical for GISH and FISH experiments. The slides with metaphase spreads were pre-treated with 10 mg/ml RNase A for 1 h at 37°C, followed by treatment with pepsin (10 mg/ml) for 15 min at 37°C. Slides were incubated in fixative solution containing 4% paraformaldehyde for 10 min at room temperature.

Double GISH used both genomic probes together (*A. duranensis* and *A. ipaënsis*-derived probes, ∼50 ng of each probe/slide). Hybridizations were performed for 14 h at 37°C, followed by 73% stringent washes, using 2x saline citrate buffer (SSC). Hybridization sites were immunocytochemically detected using the antibody anti-digoxigenin conjugated to fluorescein (Roche Diagnostics Deutschland GmbH) or by direct observation of the Cy3 fluorescence in the epifluorescence microscope Zeiss AxioPhot (Zeiss, Oberkochen, Germany). Slides were counterstained with DAPI before observation.

### SNP genotyping and data analysis

For allotetraploid plants, genetic exchange occurs mainly between chromosomes that are part of the same subgenome, *i.e.*, homologous exchanges. However, it was previously noted that a proportion of these genetic exchanges happen between different subgenomes (homeologous exchanges). In the case of peanut allotetraploids, the products of homeologous recombination form the genome configurations AAAA and BBBB (Leal-Bertioli *et al.*^2015^, ^2019^). Here, we characterized this phenomenon using (1) different generations and (2) different lineages of allotetraploid plants. Different generations consisted of seeds of selfed IpaDur1 collected from 2007 to 2016 (S_1_ to S_10_). Different lineages of allotetraploid plants from the same hybridization event comprised 15 S_2_ plants and one S_3_ plant of the tetraploid IpaDur2. All these plants from different generations of IpaDur1 and different lineages of IpaDur2 were genotyped and compared. Single plants were genotyped from each lineage/each generation.

**Figure 1:**
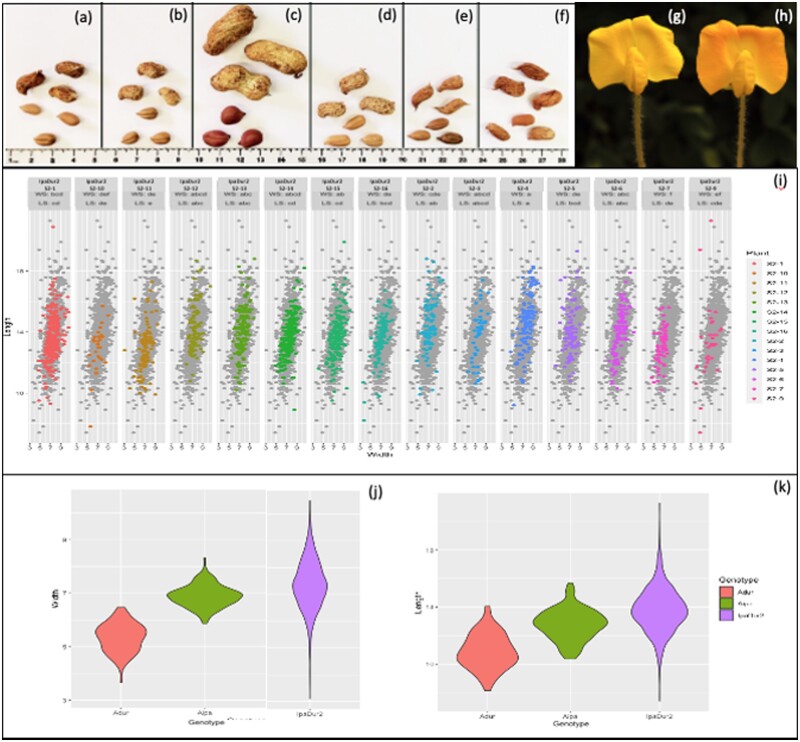
(A–F) Pods and seeds of *Arachis* genotypes used in this study. IpaDur2 (*A. ipaënsis* K 30076 **×** *A. duranensis* V 14167)^4x^ (A); IpaDur1 (*A. ipaënsis* K 30076 **×** *A. duranensis* V 14167)^4x^ (B); *A. hypogaea* subsp. *fastigiata* var. *fastigiata* “IAC Tatu-ST” (C); *A. monticola* (V 14165) (d); *A. duranensis* (V 14167) (E) and *A. ipaënsis* (K 30076) (F)*.* Scale: cm. (G, H): Yellow (G) and orange (H) flowers from the induced allotetraploid IpaDur2 (*A. ipaënsis* K 30076 **×** *A. duranensis* V 14167)^4x^. Bar: 5 mm. (I–K) Distribution of seed length (*y*-axis) and width (*x*-axis) values, in mm, across 15 IpaDur2 S_2_ plants. Distribution of entire phenotypic data of IpaDur2 plants in the background of the scatterplots and distribution of each plant is indicated by colors. Letters on top indicate significant difference among plants for seed length (LS) and width (WS) (*P*-value ≥ 0.05) (i) Violin plots show the distribution and data density for seed width and length from *A. duranensis*, *A. ipaënsis* and IpaDur2 (J, K).

Genomic DNA was extracted from young leaves using Qiagen Plant DNeasy kit (Qiagen, Germantown, MD, USA) and quantified by Qubit 4 fluorometer (Thermofisher Scientific, Waltham, MA, USA). Genotyping was performed with the Axiom Arachis SNP array v02, a 48K SNP array, designed using machine-learning models as described by Korani *et al.* (2019). The reference genomes used for the array design were of *A. duranensis* accession V 14167 and *A. ipaënsis* K 30076 (Bertioli *et al.* 2016), the same accessions and the same genetic stocks as used to make the neopolyploids used in this study. Total sizes of genome assemblies were 1211 and 1512 Mb for *A. duranensis* and *A. ipaensis*, respectively. Total repetitive contents were estimated as 61.7 and 68.5% for the *A. duranensis* and *A. ipaënsis*, respectively (Bertioli *et al.* 2016). Progenitors were genotyped as controls. Genotyping results were analyzed with the Axiom Analysis Suit (Thermofisher Scientific). Each sample was replicated at least once. Data were filtered by quality using the QC call rate >90%. The genotyping information was filtered allowing a minor allele frequency (MAF) >0.05 and 20% missing calls. Markers showing inconsistent calls from duplicates of the same sample were discarded. Data output was visualized in Microsoft Excel. Genotyping with the Axiom Array gives results which are consistent with genotyping by sequencing. For instance, we have directly compared results from the genotyping by sequencing illustrated in Bertioli *et al.* (2019) (Figure 1, left hand panels, A and B). Axiom genotyping was much more cost effective, and therefore, we used it in this study.

## Results

### Morphology

IpaDur1, produced before 2006, was propagated and maintained for nine generations of self-pollination, without intentional selection, regardless of morphological aspects, pollen viability, seed set or evidence of alterations in genome stability *i.e.*, all viable seeds were collected. All tetraploid plants had larger leaves and flowers than those of the diploid parents, but similar trailing habit and average seed size to the diploid parents (Figure 1, A–F, Leal-Bertioli *et al.* 2017). Most lineages had yellow flowers, but a few had orange flowers.

The first-generation seeds of the neopolyploid IpaDur2 (S_1_) had low-germination rate: from the over 50 seeds produced, only 16 germinated. This contrasts with the diploid parents, that have typically over 90% germination rate. The IpaDur2 plants had very similar traits to IpaDur1 (Figure 1). All S_2_ IpaDur2 plants produced yellow flowers, but in the subsequent generation, S_3_, one individual produced orange flowers (Figure 1, G and H). The number of pods produced varied widely among the individuals, ranging between one and 103 pods per plant. Seed size differed significantly among individuals according to the nonparametric Kruskal-Wallis test (Figure 1I). Levine test of variance revealed a *P*-value greater than 0.05, indicating that there was significant difference in variance between the genotypes: Variance of seed width and length of IpaDur2 was higher than *A. duranensis* and *A.* *ipaënsis* (Figure 1, J and K, Supplementary File S1). Overall, the IpaDur2 lineages we studied here had slightly more variability in seed size than IpaDur1. Both IpaDur1 and IpaDur2 plants presented a general variability in number and length of branches, main stem height, leaf color among other features, but it is difficult to assign statistical significance to this variability because each plant is an individual.

### Cytogenetic characteristics

#### Chromosome morphology

**Figure 2:**
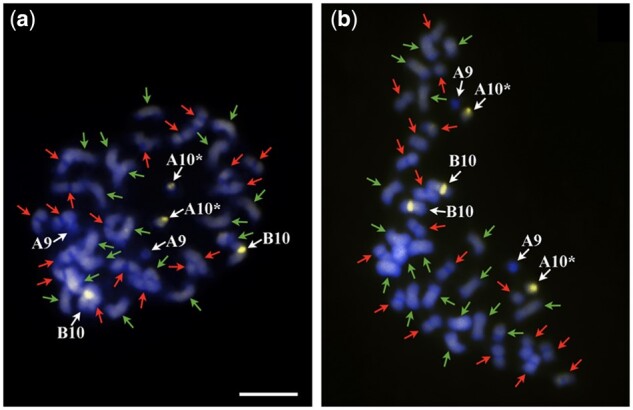
General overview of chromosomes of IpaDur2 S_2_ (A) and IpaDur1 S_10_ (B). Both genotypes showing ${\text{CMA}}_{3}^{+}$ bands (yellow) on proximal regions of cyt-A10 (A10) and cyt-B10 (B10). Chromosomes of A subgenome are indicated by green arrows (faded DAPI bands on centromeres) and chromosomes of B subgenome, by red arrows. Cyt-A10 shows secondary constriction and a short arm and a proximal segment of the long arm (*). Bar: 5 μm.

The correspondence between chromosomal pseudomolecule numbering (Bertioli *et al.* 2016) and cytogenetic numbering (Seijo *et al.* 2004, 2007; Lavia *et al.* 2008; Robledo and Seijo 2008, 2010; Robledo *et al.* 2009; Grabiele *et al.* 2012) is so far mostly unknown in *Arachis* (Bertioli *et al.* 2019). Therefore, herein, to avoid confusion, we will specify the cytogenetic nomenclature with the prefix “cyt-*X,” with an asterisk (*) referring to the type of the subgenome (A or B) and X, the corresponding number of the chromosome. The neotetraploids IpaDur2 and IpaDur1 harbored 40 chromosomes, comprising 36 metacentric and four submetacentric, which corresponds to the sum of those of the diploid parental species, *A. ipaënsis* and *A. duranensis*. In all sets of chromosomes analyzed, a small pair “A” and one satellite chromosome were identified (Figure 2, A and B). The small pair “A” corresponds to the pair of chromosomes cyt-A9* in *A. duranensis*, characterized by a remarkably high condensation level of the heterochromatin on the centromeres. In addition, the pair of chromosomes with a secondary constriction and satellite segment near to the proximal segment of the long arm of the chromosome corresponds to the pair of chromosomes cyt-A10 in *A. duranensis* (Figure 2).

#### Distribution of heterochromatic bands

IpaDur2 and IpaDur1 had conspicuous DAPI+ bands on centromeric regions of the ten pairs of chromosomes of the A subgenome while the other 10 pairs from the B subgenome lacked these bands. These DAPI+ banding patterns in the induced tetraploids were indistinguishable from *A. hypogaea* and *Arachis* *monticola* and the same as expected for the sum of the diploid progenitors. This indicates the conservative organization of these chromosomal structures after allopolyploidization, in lineages formed at different times, induced and spontaneous (Figure 2).

CG-rich repetitive heterochromatic regions in the DNA that displayed ${\text{CMA}}_{3}^{+}$ bands were observed on the proximal regions of chromosomes cyt-A10 and cyt-B10 of both IpaDur2 S_2_ and IpaDur1 S_10_ (Figure 2, A and B, respectively), corresponding to the sum of the bands of the parental species *A. duranensis* and *A. ipaënsis* (Nascimento *et al.* 2018). However, this pattern differed from the other two allotetraploids, *A. hypogaea* [with ${\text{CMA}}_{3}^{+}$ bands on cyt-A2, cyt-A10, cyt-B3, cyt-B7, and cyt-B10, (Nascimento *et al.* 2018)] and *A. monticola* [with ${\text{CMA}}_{3}^{+}$ bands only on cyt-A2 and cyt-A10 (Nascimento *et al.* 2020)]. These differences in ${\text{CMA}}_{3}^{+}$ band number and distribution may be due to changes in DNA sequence, or in the organization of the DNA that reduced binding of the fluorophore.

#### Affinity to progenitor genomes by GISH

**Figure 3:**
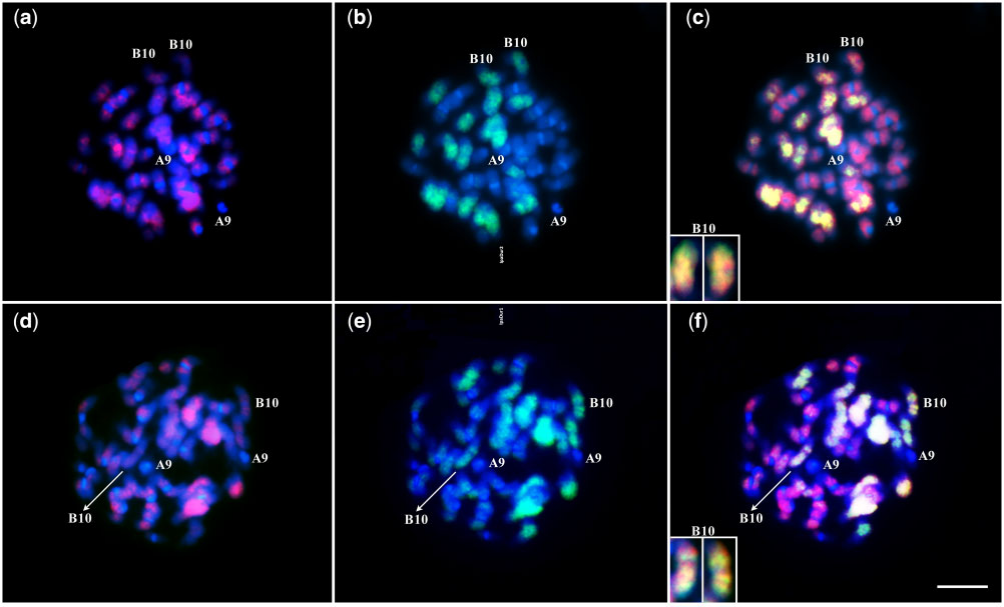
Double GISH in IpaDur2 S_2_ (A–C) and IpaDur1 S_10_ (D–F) using simultaneously, *A. duranensis* (*red*) and *A. ipaënsis* (*green*) probes, followed by DAPI counterstaining (blue). Note the preferential affinity of *A. duranensis* probe (*red*) to chromosomes of the A subgenome showing DAPI+ bands on centromeres (A, D), and of *A. ipaënsis* probe (*green*) to chromosomes of the B subgenome, lacking DAPI bands (B, E). *A. duranensis* probe (*red*) also show some hybridization to chromosomes of the B subgenome, indicating genome inversion A to B. Many chromosomes have yellow signals, that result from the overlapping green and red sites indicating DNA sequences that are shared between A and B subgenomes, observed. Image insets of cyt-B10 show mosaic pattern of hybridization, indicating recombination between A and B subgenomes in IpaDur2 (C) and IpaDur1 (F). Bar = 5 μm.

GISH signals were present on all chromosomes of IpaDur1 and IpaDur2, for the corresponding subgenomes A and B. Double GISH, using both genomic probes on IpaDur2 (Figure 3, A–C) and IpaDur1 chromosomes (Figure 3, D–F) confirmed the preferential hybridization of the chromosomes of the *A. duranensis* probe to the A subgenome chromosomes (red, Figure 3, A and D) and of the *A. ipaënsis* probe to the B subgenome chromosomes (green, Figure 3, B and E) similarly to what has been shown for *A. hypogaea* and *A. monticola* (Seijo *et al.* 2007; Nascimento *et al.* 2018). Hybridization signals were weaker at chromosome terminal regions. However, for most chromosomes overlapping hybridization signals were observed, *i.e.*, both probes hybridized to the same DNA region (yellow, Figure 3, C and F), indicating DNA sequences that are shared by subgenomes A and B. On both genotypes, there were mosaic hybridization patterns on cyt-B10 (green signal interspersed with red signals), indicating recombination between A and B subgenomes (Figure 3, C and F). Cyt-B10 also showed extensive yellow signals, indicating “invasion” of A alleles onto B genome chromosome.

#### Distribution of rDNA loci

The number, size, and position of the 5S rDNA loci on chromosomes of IpaDur2 and IpaDur1 were identical (Figure 6 and Nascimento *et al.* 2018). The loci were situated on the proximal region of chromosomes cyt-A3 and cyt-B3, along the short arms, as were observed in each diploid species, *A. duranensis* and *A. ipaënsis*, respectively, thus representing an additive character in both neopolyploids.

**Figure 4:**
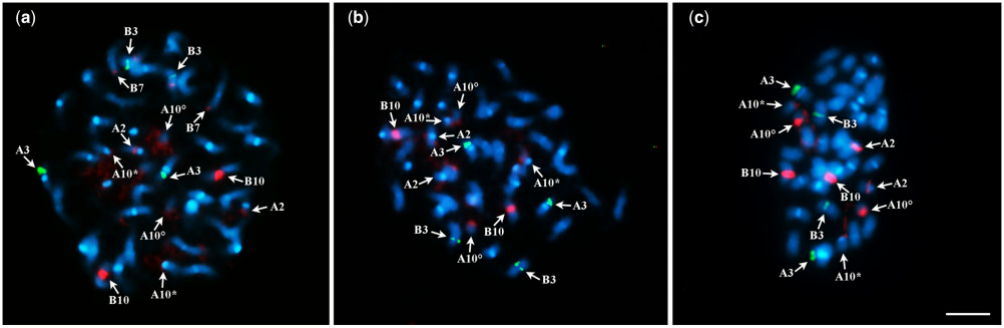
Double FISH on somatic metaphases of IpaDur1 S_10_ (A) and IpaDur2 S_2_ (B, C) after double FISH using simultaneously, 5S ribosomal DNA (rDNA) (*green*) and 45S rDNA (*red*) probes, followed by DAPI counterstaining. Twenty chromosomes of the A subgenome have DAPI+ bands on centromeres (*light blue*). The 5S rDNA loci (*green*) are detected on cyt-A3 (A3) and cyt-B3 (B3) on both genotypes on all preparations (A–C). 45S rDNA (*red*) are detected on IpaDur1 on cyt-A2 (A2), cyt-A10 (A10), cyt-B10 (B10) (A). On IpaDur2, most metaphases show the same pattern (B), whereas some metaphases show two extra sites, on cyt-B3 (B3) and cyt-B7(B7) (C). Cyt-A10 short arm and proximal segment of the long arm (*) and satellite (°). Bar: 5 μm.

**Figure 5:**
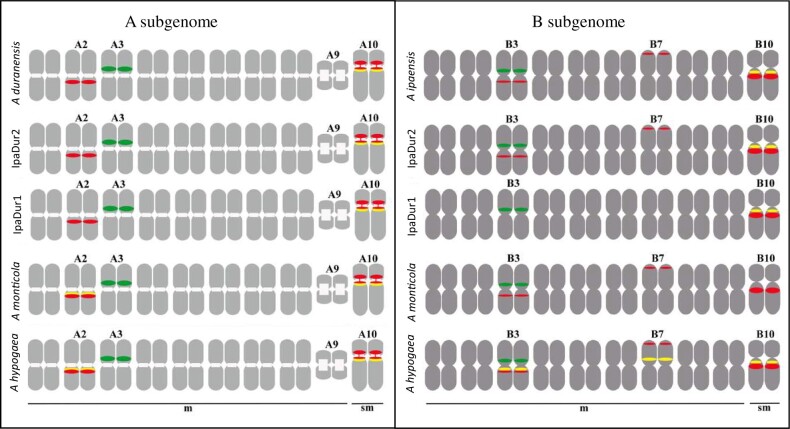
Schematic diagram of some *Arachis* karyotypes showing variability in chromosome morphology; centromeres (m, metacentric; sm, submetacentric); DAPI^+^ banding (white), ${\text{CMA}}_{3}^{+}$ bands (yellow), and 5S (green), and 45S (red) rDNA loci in *A. duranensis* V 14167, *A. ipaënsis* K 30076, IpaDur2, IpaDur1, *A. monticola* V 14165, and *A. hypogaea* “IAC-Tatu-ST” (from Nascimento *et al.* 2020). Variability is detected on cyt-A2, cyt-B3, cyt-B7, and cyt-B10. Only the most common configuration of IpaDur2 is presented.

The 45S rDNA loci in IpaDur1 S_10_ (Figure 4A) consisted of two loci from *A. duranensis* (on cyt-A2 and cyt-A10) and just one locus out of three loci originally present in *A. ipaënsis* (on cyt-B10) thus not being an additive character (this was also observed in Nascimento *et al.* 2018). In IpaDur2 S_2_, two patterns of 45S rDNA hybridization were observed: three of these loci were detected in all metaphases (proximal regions of cyt-A2 and cyt-B10 and near the secondary constriction of cyt-A10); the remaining two loci (proximal regions of cyt-B3 and cyt-B7 terminal regions, Figure 4C), on some individuals, presented weak or no detectable hybridization signals on others (Figure 4B). Notably, this variability was observed not only between individuals, but also between cells of the same individual. Cyt-B3 showed co-localization of 5S and 45S loci, whilst cyt-A10 showed the largest rDNA 45S site (green), with an intense signal on the secondary constriction, and a thread-like aspect, characteristic of a NOR (Nucleoli Organizing Region). *A.* *hypogaea* and *A. monticola* have five loci easily detected on the proximal region of the long arm of cyt-A2, cyt-A10, and cyt-B10, proximal region of short arm of cyt-B3 and terminal region of the short arm of cyt-B7, corresponding to the sum of the two loci from *A. duranensis* and three from *A. ipaënsis* (Nascimento *et al.* 2020). All these results are summarized in Figure 5. The karyotypes of *A. hypogaea* subsp. *fastigiata* var. *fastigiata* “IAC Tatu-ST,” *A. monticola* (V 14165), *A. duranensis* (V 14167), and *A. ipaënsis* (K 30076), described previously (Nascimento *et al.* 2018, 2020) were included in Figure 5 to enable comparison.

### Genome-wide SNP analysis of genome stability of induced allotetraploids

It is known that for allotetraploid plants, genetic exchange occurs mainly between chromosomes that are part of the same subgenome, *i.e.*, homologous exchanges. However, it was previously noted that a proportion of these genetic exchanges in allotetraploid *Arachis* happen between different subgenomes (homeologous exchanges) (Leal-Bertioli *et al.* 2015; Clevenger *et al.* 2017; Bertioli *et al.* 2019).

**Figure 6:**
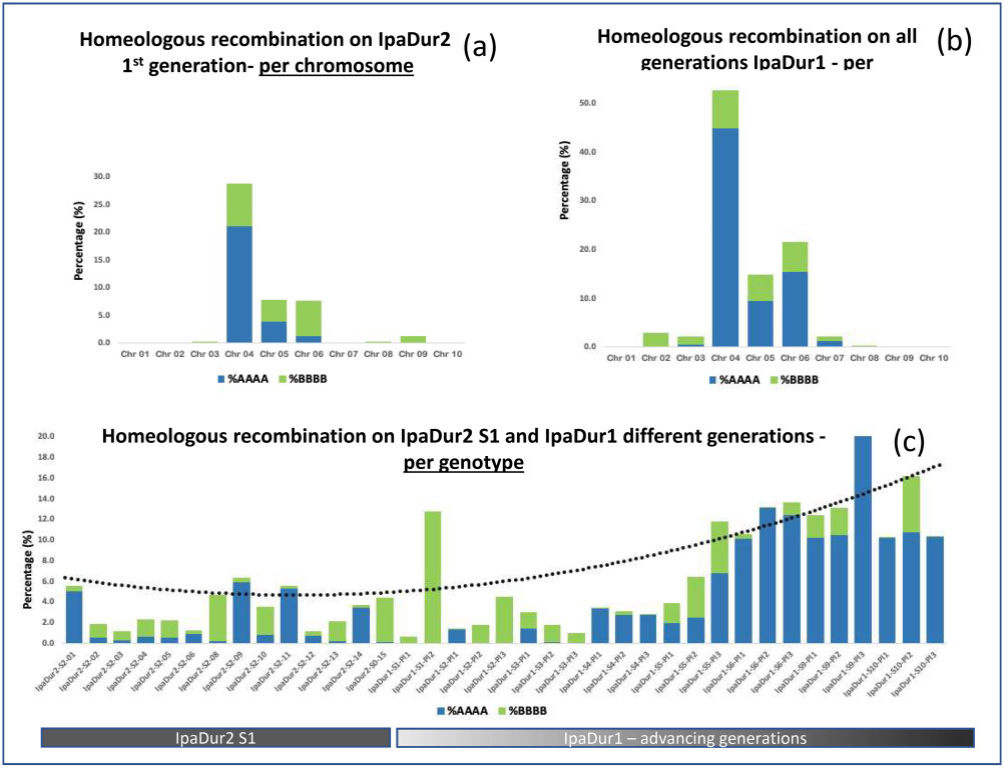
Histograms showing the total percentage of homoeologous recombinations: (A) in each chromosome of IpaDur2 first generation; (B) in each chromosome of IpaDur1, an average of six different generations; and (C) on all individuals of all generations. Blue are the conversions from AABB to AAAA, and green, conversions to BBBB. Note that conversion to AAAA is prevalent, Chromosome 4 has most homoeologous recombinations and that there is a tendency for individuals of later generations to accumulate more homoeologous recombinations.

Here, we investigated genome-wide variation in progeny from the two polyploidy events, IpaDur1 and IpaDur2, in different generations: 15 different lines of the first generation (S_1_) of IpaDur2 (BBAA genome) and 23 individuals of eight generations of the previously obtained induced allotetraploid IpaDur1 (BBAA genome), using the 48K Affymetrix chip (Korani *et al.* 2019). Four thousand two hundred and sixty-three polymorphic loci were detected between *A. duranensis* (AA genome) and *A. ipaënsis* (BB genome) and used for genome-wide analysis. Because the current analysis was investigating the autotetraploid-like behavior (recombination between homeologs), the loci were ordered according to the ten chromosomal pseudomolecules (chr) of *A. ipaënsis*. There was significant variation between IpaDur1 and IpaDur2 individuals (Figure 6). The large majority of loci presented the expected AABB composition, AA from *A. duranensis* and BB from *A. ipaënsis*. However, all chromosomes presented some loci with the composition AAAA or BBBB, indicating homeologous recombination. Two patterns of homeologous recombinations were observed: loci that were interspersed throughout the chromosomes and, the most prevalent, loci that occur in continuous segments, or blocks (*e.g.*, IpaDur2-S_2_-pl11 chr A04/B04, Supplementary File S2). The interspersed homoeologous loci can be plausibly explained by gene conversion whilst blocks can be plausibly explained by tetrasomic meiotic recombination. The overall rate of homeologous recombination detected for IpaDur2 S_2_ was 3.32%, and for all generations of IpaDur1 was 7.76% (Figure 6A). However, this is likely to be underestimated because with SNP chip genotyping is not possible to detect genome changes that result from balanced homeologous exchanges. Homeologous exchange in blocks were mostly detected at the distal parts of the pseudomolecules while interspersed loci were detectable throughout the body of chromosomes (Supplementary File S2).

The chromosomes A04/B04 (42.74%), A06/B06 (16.02%), and A05/B05 (12.14%), which all show a high level of collinearity (Bertioli *et al.* 2019), had the highest frequency of homoeologous recombination for all induced allotetraploids: changes in these chromosomes occurred in all 38 lineages that were randomly chosen for analysis (Figure 6, A and B, Supplementary File S2). Using GISH cytogenetic analysis, chromosomes cyt-B10 of IpaDur1 and IpaDur2 showed mosaic hybridization patterning, suggesting that this is the chromosome with the highest level of genomic exchange between subgenomes, and prevalent invasion of A alleles onto the B subgenome (Figure 3). Because genetic and cytogenetic analyses agree, we can plausibly suggest that cyt-B10 corresponds to chromosomal pseudomolecule B04.

There was an overall similar tendency for conversion to AAAA (1.79%) and to BBBB (1.53%) across all chromosomes in IpaDur2 individuals. On IpaDur1, however, there was a much higher rate of conversion to AAAA (5.69%) than to BBBB (2.05%). In all 38 individuals, from both allotetraploids and different generations, exchanges were generally asymmetrical—most genotypes had severe bias to either AAAA or BBBB. The exceptions were IpaDur2 S_2_-12 (58.6% AAAA/41.4% BBBB) and IpaDur1 S_7-_3 (57.9% AAAA/42.1% BBBB). There was a strong bias towards AAAA (4.77% compared to 2.03% BBBB). Previously it has been shown that *A. hypogaea* has overall bias towards BBBB, but that this tendency was reversed to AAAA towards the chromosome ends (Bertioli *et al.* 2019).

For IpaDur2 S_2_, homoeologous recombination ranged from 1.23% to 6.36% throughout the whole genome (average 3.32%). For IpaDur1, where we were able to access different generations, a much wider range was observed: 0.7 (IpaDur1 S_3_-1) to 20.08% (IpaDur1S_11_-3). Homeologous recombination tended to increase over generations (Figure 6C).

## Discussion

Peanut is an allotetraploid of recent origin with an AABB type genome (2*n* = 4x = 40). It arose from one, or a very few, events of hybridization between the diploid species *A. duranensis* and *A. ipaënsis* followed by spontaneous tetraploidization (Kochert *et al.* 1996; Seijo *et al.*2007; Bertioli *et al.*^2016^, ^2019^). Here, we studied induced neotetraploids obtained from the sequenced accessions of the progenitor species (V 14167 and K 30076) in two distinct events (Favero *et al.* 2006; Bertioli *et al.* 2019). This gave us the opportunity to observe independently generated lineages and different generations of the same lineage. The first generation of both lineages (IpaDur1 and IpaDur2) showed low germination rate, and highly variable fertility, with some individuals producing no pods at all. However, fertility in later generations recovered. This is consistent with the suggestion made by Ramsey and Schemske (2002) that newly formed allopolyploids have reduced fertility because of unbalanced gametes, but that, genetics permitting, lineages which form balanced gametes are selected. A sufficient level of fertility clearly being a condition for young polyploids to form viable sexually reproducing lineages in the long term. In this study, we show that the induced allotetraploid lineages that advanced in generations exhibited enhanced diversity in seed size and pod characteristics, flower color, and genome configurations (Figures 1 and 6, Supplementary Materials 2). A similar rapid diversification following the spontaneous formation of the polyploid species, *A. monticola* and *A. hypogaea*, provides a highly plausible mechanism which allowed an allotetraploid, with such narrow genetic origin, to diversify phenotypically and be favored in domestication over much more genetically diverse diploid species.

For several years, it was naïvely assumed that these newly induced allotetraploids had (1) a genome that was the sum of the A and B subgenomes in equal parts, forming an AABB composition in all loci, and (2) followed diploid genetics: that is, homologous chromosomes would exclusively pair at meiosis. However, as the tools for genetic mapping advanced, and denser datasets became available, we discovered that both assumptions were inaccurate. Genome regions with deviant compositions, AAAA or BBBB were detected, demonstrating that homoeologous recombination was surprisingly common (Leal-Bertioli *et al.*^2015^, ^2018^; Clevenger *et al.* 2017). Although trivalent and tetravalent chromosome associations had been previously observed during meiosis of cultivated peanut in cytogenetic studies (Husted 1936; Smartt *et al.* 1978; Singh and Moss 1982; Wynne *et al.* 1989), the occurrence of homoeologous recombination in genetic studies had gone almost unnoticed before (Leal-Bertioli *et al.* 2015). The role of homoeologous recombination in the evolution and domestication of several allopolyploids has been well documented, in oilseed rape (*Brassica napus*; Sharpe *et al.* 1995; Chalhoub *et al.* 2014; Hurgobin *et al.* 2018), and in other species, including quinoa (*Chenopodium quinoa*; Jarvis *et al.* 2017), and tobacco (*Nicotiana tabacum*; Chen *et al.* 2018). Homoeologous recombination is now thought to not only alter gene dosage of large chromosomal segments but also to provide a mechanism for evolutionary novelty in transcript and protein sequences in nascent allopolyploids, including the formation of intergenomic “recombinant” proteins (Zhang *et al.* 2020). In this study, we observed the presence of homoeologous recombination in several chromosomes and its consequences at the cytogenetic level.

In 2008, under the scope of the Generation Challenge Programme (www.generationcp.org), seeds of IpaDur1 were distributed to different research groups around the world (in Senegal, France, USA, Argentina), becoming a public resource. With this, independent studies were performed by different groups, with occasional discrepancy in some results. An example is the contrasting results of Nascimento *et al.* (2018) and Seijo *et al.* (2018) who did cytogenetic analyses on what was assumed to be the same genotype, IpaDur1. Both groups observed large macrostructural stability of karyotype, however differences were observed in the number of detectable 45S loci. In the work here, we found this very same divergence between different events of tetraploidization of the newly formed IpaDur2, and even between different cells of roots from the same plant: some metaphases showed five, some showed only three 45S rDNA loci (Figure 4). This recurring inconsistency in the number of 45S rDNA sites detected indicates high genome instability in these distinct lineages distributed to both groups. One possibility is that 45S rDNA tandem repeats were lost or reduced in size in cyt-B3 and cyt-B7 in both IpaDur1 and IpaDur2; another is the remodeling of chromatin (Liu and Wendel 2003; Neves *et al.* 2005; Chiavegatto *et al.* 2019) that altered probe hybridization or fluorescence detection (also see Gao *et al.* 2021). These chromosomal changes are consistent with those observed in other recently formed allotetraploids (Xiong *et al.* 2011; Chester *et al.* 2015) but contrasts very strongly to the higher cytogenetic stability generally observed in polyploid crops, including the natural allotetraploids, *A. hypogaea* and *A. monticola* (Nascimento *et al.* 2018; Bertioli *et al.* 2019). This corroborates the inference that in early generations high instability naturally occurs, but in later generations, genome becomes stabilized by suppression of homeologous exchange and/or selection of lines with lower levels of chromosome rearrangements (Cifuentes *et al.* 2010; Zhang *et al.* 2020). This initial instability would provide phenotypic variation upon with artificial selection could act, thus offering the opportunity for niche expansion in the new allotetraploid species. In a recent review, Van de Peer *et al.* (2021) also highlighted that the response to biotic and abiotic stresses are important in determining the establishment and success of new polyploids. Such stresses are likely to be encountered in the new ecological niches of domestication and the preferential selection of tetraploid *Arachis* over diploid could result from their more varied and/or improved responses (phenotypic plasticity) awarded by polyploidy.

In addition to cytogenetic characterization, we addressed genome changes by assaying the dosage of single nucleotide polymorphisms (SNPs) which differentiated the two diploid progenitor species in the DNA of 39 lineages derived from two events of polyploidization. In the early generations derived from IpaDur2, we found that between 1.23 and 6.36% of the genome had been impacted by homeologous recombination. That is, the genome had converted from an AABB configuration to AAAA or BBBB. Although variable between lineages, the results with IpaDur1 indicate that this percentage increased in later generations, a characteristic of the polyploid ratchet (Figure 6; Gaeta and Pires 2010). The phenotypic variability observed were differences in germination rate and fertility in early generations, and flower color and seed size in later generations. These can be very plausibly explained by genomic instability. Homoeologous recombination was more frequent in some chromosomes than others. From the sixth generation onwards, for the lineages studied, the chromosome A04/B04 SNP alleles assayed entirely converted to AAAA, a type of “total collapse” (Figure 6A, Supplementary File S2). Notably, however, the chromosomal composition of these lines as observed at the cytogenetic level remained 10 A chromosome pairs, and 10 B chromosome pairs, but with one pair of chromosomes (cyt-B10) showing an interspersed mosaic patterning in GISH. This genomic “collapse” could also account for the viability loss observed in some lineages at S_9_ and S_10_ generations. If, as seems likely, similar genetic instability was present at the origin of *A. hypogaea* and *A. monticola—*how did they regain stability?

Genome instability has, in the past, led some authors to consider polyploidy an “evolutionary dead end” (*e.g.*, Mayrose *et al.* 2011). However, it is well established that polyploids can survive and flourish with controlling mechanisms to ensure some level of genome stability during regular meiotic division, and consequent fertility. Regular segregation of chromosomes during meiosis requires a restriction of pairing between homoeologous chromosomes to avoid configurations that lead to the production of unbalanced and aneuploid gametes, aneuploid progenies, chromosome rearrangements and reduced fertility (Benavente *et al.* 2001; Sánchez-Morán *et al.* 2001; Ramsey and Schemske 2002). To date, only two mechanisms of genome stabilization in allopolyploids have been clearly defined: *differential affinity* and *pairing regulators*. *Differential affinity* [concept by Darlington (1928)] is when differences between the complementary homeologous chromosomes (structural changes and DNA sequence divergence) preclude homoeologous chromosome pairing. In fact, a study of four wheat allotetraploids showed that instability caused by homoeologous exchange were nonrandomly distributed along the chromosomes. Density of homoeologous exchanges increased on both arms along the centromere–telomere axis, corresponding to genomic regions with higher gene density, higher DNA similarity, lower DNA methylation level, and higher chromatin accessibility (Zhang *et al.* 2020). *Pairing regulators* is when genetic systems regulate homoeologous pairing. Such regulators have been identified in several allopolyploid species, such as wheat, where the gene *Ph1* has been shown to contribute to genetic stabilization of wheat by suppressing homoeologous pairing at Meiosis I. In its absence, hexaploid wheat is not able to form stable bivalents (Riley and Chapman 1958; Riley 1960; Griffiths *et al.* 2006). In addition, over eight genes have been implicated with genome stabilization in the autotetraploid *Arabidopsis arenosa* (Morgan *et al.* 2020). The relative importance of the genes contributing to the cytological diploidization of polyploids is presumably generally greater with more closely related homoeologous genomes. However, it is not trivial to disentangle the relative effects of differential affinities and pairing regulator genes (Jenczewski and Alix 2004, Mason and Wendel 2020). In cultivated peanut, there is evidence for both. First, differential affinity may have been enhanced by chromosomal deletions and rearrangements following polyploidy. In particular, it is notable that the inversion in chromosome A05 relative to the sequenced diploid ancestor is immediately adjacent to the largest tetrasomic genome conformation in the tetraploid genome (Bertioli *et al.* 2019)*.* It is very plausible that this inversion, by disrupting collinearity, could have hindered extensive homoeologous exchange, stabilizing chromosomes A05/B05 from total collapse. Second, the existence of pairing regulators in *Arachis* is implied by the QTLs associated with homoeologous recombination frequency identified in Leal-Bertioli *et al.* (2015).

**Figure 7:**
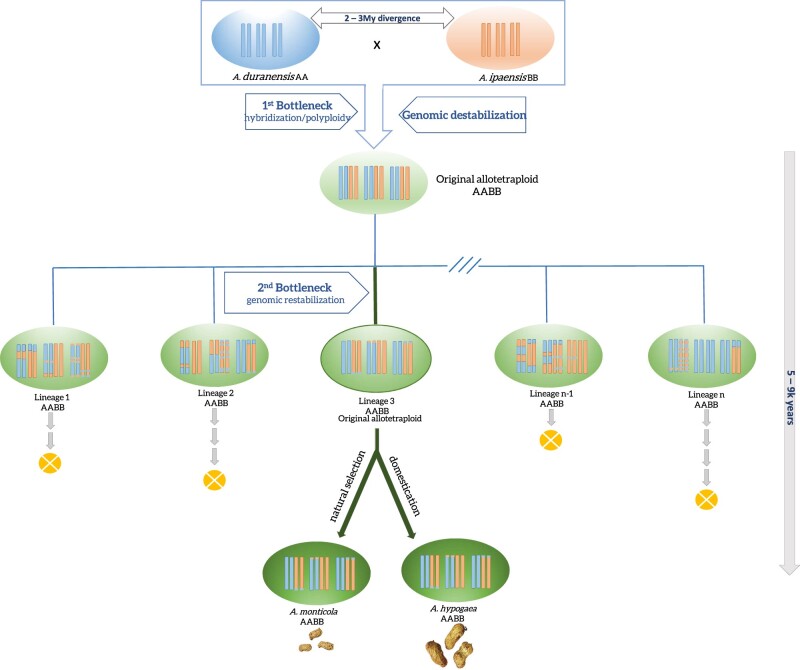
Schematic representation of the origin and evolution of the two spontaneously formed allotetraploids, the wild *A. monticola* and the cultivated peanut, *A. hypogaea*. The diploid *A. duranensis* crosses with *A ipaënsis* to generate a sterile diploid hybrid, that undergoes natural polyploidization. The rarity of this event and the barrier that the ploidy causes characterize the first bottleneck. Lineages originated from the nascent allotetraploid have significant genomic instability (here observed by genotyping and cytogenetics), abnormal chromosome pairing, causing low fertility. Most lineages collapse, except the ones that have efficient mechanisms in place that homoeologous exchange. This survival of only a few lineages represents the second bottleneck in evolution of peanut.

It has long been accepted that initial genome duplication event at the origin of peanut created a bottleneck between the domesticated and its diploid wild relatives (Figure 7). We had previously suggested that genetic deletions and exchange between A and B subgenomes generated variation that helped to favor the domestication of *A. hypogaea* over its diploid relatives (Bertioli *et al.* 2019). Here, we further conclude a scenario for the origin of *A. hypogaea* and *A. monticola*: after the initial polyploidization, the resulting individuals underwent a period of genomic instability, generating high phenotypic variability, ranging from individuals with inability to produce seed to those with stabilizing genetic variants. We propose that lineages fit for domestication captured phenotypic variations favorable to cultivation but also stronger genetic mechanisms for limiting genome instability which favored their long-term survival.

## Data availability

All data are in the Supplementary Files. Diploid progenitor accessions are available at the USDA-PGRCU (Plant Genetic Resources Conservation Unit at the United States Department of Agriculture) under accession numbers PI 468322 and PI 692197. Allotetraploids may be obtained from the authors under request. Supplementary material is available at figshare: https://doi.org/10.25387/g3.14696724.
